# Comparison of Preoperative Magnetic Resonance Imaging and Intraoperative Frozen Section Analysis with Final Pathological Outcomes in the Assessment of Myometrial Invasion in Endometrial Cancer Cases

**DOI:** 10.3390/diagnostics15212799

**Published:** 2025-11-05

**Authors:** Tuba Metin Çakır, Fatma Ceren Güner, Elif Iltar, Can Dinç, Ömer Faruk Öz, Tayup Şimşek

**Affiliations:** 1 Department of Obstetrics and Gynecology, Akdeniz University, Antalya 07070, Turkey; candinc00@hotmail.com (C.D.); dromerfarukoz@gmail.com (Ö.F.Ö.); 2 Department of Obstetrics and Gynecology, Division of Gynecologic Oncology, Akdeniz University, Antalya 07070, Turkey; fcereng@gmail.com (F.C.G.); dktr_elif@hotmail.com (E.I.); tsimsek@akdeniz.edu.tr (T.Ş.)

**Keywords:** endometrial cancer, frozen section, magnetic resonance imaging, myometrial invasion

## Abstract

**Background**: The aim of this study is to compare the concordance of Preoperative Magnetic Resonance Imaging (MRI) and Intraoperative Frozen Section Analysis, widely used worldwide for Endometrial Cancer (EC), with final pathology results, to calculate their sensitivity and specificity, and to evaluate their diagnostic agreement with final pathology results. Positive predictive values for both MRI and frozen section analysis will also be calculated. **Methods**: In this retrospective cohort study, patients diagnosed with Endometrioid-Type Endometrial Cancer at the Gynecologic Oncology Surgery Department of Akdeniz University Hospital between January 2020 and December 2023 underwent preoperative MRI to assess the depth of myometrial invasion and intraoperative frozen section examination for surgical staging. The results of both methods were compared with the final pathology reports. **Results**: A total of 88 patients were included in the study. Patient ages ranged from 34 to 80 years, with a mean age of 57.57 years (SD: 9.65), predominantly in the middle-aged and older population. In the assessment of myometrial invasion depth, MRI demonstrated a sensitivity of 81.6% and a specificity of 88%, while frozen section analysis showed a sensitivity of 73.7% and a specificity of 98.0%. **Conclusions**: In our study, preoperative MRI demonstrated similar sensitivity and specificity to intraoperative frozen section analysis in determining the depth of myometrial invasion in cases of Endometrioid-Type Endometrial Cancer. Therefore, when intraoperative frozen section analysis is not available, MRI findings may assist surgical planning, particularly in centers where frozen section is limited.

## 1. Introduction

Endometrial cancer is the fourth most common malignancy among women in resource-rich countries [1]. Although it is typically regarded as a postmenopausal disease, approximately 15% of cases are diagnosed in premenopausal women, with some being under the age of 40 at the time of diagnosis [2]. Endometrial cancer generally has a favorable prognosis, with the majority of patients diagnosed at an early stage. When all stages are considered collectively, the overall five-year survival rate has been reported to be approximately 80% [3]. Most patients present with endometrioid histology, and the five-year survival rate for well-differentiated, stage I endometrioid-type cancers approaches 90% [1,4,5]. The most common presenting symptom is abnormal uterine bleeding or postmenopausal bleeding, and because bleeding often occurs early in the disease course, most cases are detected at an early stage [6]. A preoperative diagnosis of endometrial cancer is typically established via dilation and curettage or endometrial biopsy.

The staging of endometrial cancer is based on the surgical staging system established by the International Federation of Gynecology and Obstetrics (FIGO), first introduced in 1988 and subsequently revised in 2009 and most recently in 2023. This system incorporates various parameters, including histologic subtype, tumor grade, depth of myometrial invasion, tumor size, presence of lymphovascular space invasion (LVSI), cervical stromal involvement, ovarian metastasis, pelvic and para-aortic lymph node metastases, distant organ metastases, and molecular classification. The prognosis of endometrial cancer depends on these factors—particularly stage, depth of myometrial invasion, cervical involvement, LVSI, and histologic grade [7]. However, the most significant prognostic indicators are histologic grade and the depth of myometrial invasion. As tumor grade increases, the likelihood of deep myometrial invasion and extrauterine spread rises, while overall survival rates decline.

Radiological methods such as ultrasonography, computed tomography, and magnetic resonance imaging (MRI) can be used for the preoperative assessment of myometrial invasion in endometrial carcinoma [8]. Although transvaginal ultrasonography is often the initial diagnostic modality due to its accessibility, cost-effectiveness, and high sensitivity, MRI is considered the most accurate imaging technique for evaluating myometrial invasion preoperatively in patients with endometrial cancer, particularly those under the age of 60 [9,10,11]. MRI demonstrates high specificity in the preoperative assessment of deep myometrial invasion, cervical stromal involvement, and lymph node metastases, owing to its superior soft tissue resolution [12]. MRI is also advantageous in evaluating local pelvic spread and peritoneal metastasis [13]. Furthermore, it is utilized post-treatment to investigate suspected local recurrences and to determine the location and extent of such recurrences [14]. In some centers, intraoperative frozen section analysis may not always be available, making MRI an important preoperative alternative for assessing myometrial invasion and guiding surgical planning. In recent years, MRI has increasingly been recommended for image-guided adaptive brachytherapy, reflecting its expanding role in clinical practice. This approach enables optimized tumor targeting while delivering appropriate radiation doses to organs at risk [15].

The standard treatment for endometrial cancer (EC) is total hysterectomy with bilateral salpingo-oophorectomy. Depending on the depth of myometrial invasion and the histologic type of the tumor, lymphatic staging may be performed [16]. In certain histologic subtypes, particularly serous carcinoma, undifferentiated endometrial carcinoma, and carcinosarcoma, staging procedures also include infracolic omentectomy due to the high risk of microscopic omental metastases [17].

Intraoperative frozen section analysis plays a critical role in surgical decision-making by assessing the depth of myometrial invasion, tumor size, and cervical involvement during surgery. When the resected uterine specimen is sent for final pathological examination, concordance between preoperative diagnosis and final pathology has been reported to range between 60% and 80% [18,19]. Therefore, employing a diagnostic method that can accurately predict tumor spread and pathological characteristics preoperatively can guide treatment strategies and minimize intraoperative complications. This, in turn, may reduce the need for extensive surgical procedures, improve patient quality of life, and help avoid the complications and high costs associated with radical surgery.

The aim of our study is to compare the concordance of preoperative MRI, a widely used diagnostic tool for endometrial cancer, and intraoperative frozen section analysis with final pathology results. We aim to calculate the sensitivity and specificity of both methods and to evaluate their diagnostic agreement with final pathology results.

## 2. Methods

### 2.1. Study Population and Patient Selection

In this retrospective cohort study, data from patients diagnosed with endometrioid-type endometrial cancer between January 2020 and December 2023 at the Division of Gynecologic Oncology Surgery, Akdeniz University Hospital (Antalya, Türkiye), were analyzed. During the study period, a total of 140 patients with surgically staged endometrial carcinoma were reviewed, and 88 patients who met the inclusion criteria were included in the final analysis. This was a retrospective cohort study using non-probabilistic sampling, including all eligible patients who met the inclusion criteria. Staging procedures were performed according to institutional protocols and multidisciplinary board decisions to reduce potential selection bias. The study included patients whose diagnoses were confirmed via endometrial biopsy, dilation and curettage, or following hysterectomy, and who subsequently underwent total hysterectomy with bilateral salpingo-oophorectomy for surgical staging. Additional staging procedures, such as pelvic and/or para-aortic lymph node dissection, omentectomy, and peritoneal cytology, were performed when indicated.

Exclusion criteria included absence of preoperative MRI, lack of intraoperative frozen section analysis, and incomplete medical records. In cases where sentinel lymph node mapping was successfully performed, intraoperative frozen section analysis was not conducted; therefore, these patients were also excluded from the study. Non-endometrioid histologic subtypes were excluded to maintain homogeneity, as they demonstrate distinct biological behavior and prognostic profiles.

Both laparotomy and minimally invasive (laparoscopic) approaches were used for staging surgeries, with the choice of surgical method determined by the patient’s condition and the surgeon’s preference.

**Figure 1:**
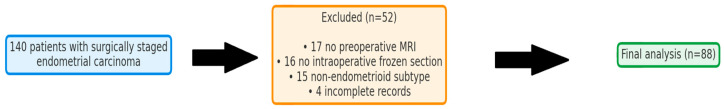
Flow diagram showing the process of patient selection and exclusion criteria.

A simplified flow diagram summarizing the patient selection and exclusion process was prepared to enhance clarity (Figure 1).

### 2.2. MRI Protocol

Magnetic resonance imaging was used for the preoperative assessment of endometrial tumors in this study. The imaging protocol included T1- and T2-weighted sequences as well as dynamic contrast-enhanced imaging, with axial, coronal, and sagittal planes acquired.

MRI examinations were performed using a 1.5-Tesla scanner (MAGNETOM Aera, Siemens Healthineers, Erlangen, Germany) equipped with a pelvic phased-array coil. Axial and sagittal T2-weighted Turbo Spin Echo (TSE) sequences were obtained with a slice thickness of 4 mm, an interslice gap of 1 mm, and a matrix size of 320 × 320. Dynamic contrast-enhanced T1-weighted sequences were acquired in the axial plane following intravenous administration of gadolinium-based contrast material (Gadovist®, Bayer AG, Leverkusen, Germany). Diffusion-weighted imaging (DWI) sequences with b-values of 0 and 800 s/mm^2^ were obtained in the sagittal plane to assess lesion conspicuity and myometrial invasion depth.

The depth of myometrial invasion was evaluated on dynamic contrast-enhanced T1-weighted images, while myometrial thickness was assessed on sagittal T2-weighted images. Tumor dimensions were measured in three dimensions using axial and sagittal T2-weighted sequences. This imaging protocol, comprising Turbo Spin Echo (TSE), T2-weighted imaging, and dynamic contrast-enhanced T1 sequences, enabled accurate preoperative evaluation of tumor extent and contributed to optimal surgical planning.

MRI examinations were interpreted by board-certified radiologists within the Department of Radiology. Reports were retrieved from the Picture Archiving and Communication System (PACS, Sectra AB, Linköping, Sweden), and radiologists did not have access to intraoperative or final pathology results at the time of MRI interpretation. Equivocal findings followed routine senior review/consensus prior to report finalization.

### 2.3. Intraoperative Frozen Section Analysis and Definitive Histopathological Evaluation Protocol

During surgery, the frozen section technique was employed to assess the extent of tumor spread. Initially, the tumor’s location and size were evaluated macroscopically, followed by the collection of 2 to 3 full-thickness tissue samples aimed at determining the depth of myometrial invasion. In the collected specimens, myometrial invasion was classified as <50% or ≥50% relative to the total myometrial thickness. The group with <50% myometrial invasion was defined as superficial invasion, while the group with ≥50% myometrial invasion was defined as deep invasion.

All intraoperative frozen section analyses were performed by pathologists from the same pathology department, following the institutional protocol to ensure consistency in evaluation.

In addition, adjacent structures, including the uterine serosa, ovaries, fallopian tubes, and cervical stroma, were examined for possible invasion. In suspicious cases, additional tissue samples were obtained for detailed pathological assessment. This approach allowed for individualized surgical planning tailored to the extent of disease observed intraoperatively.

### 2.4. Statistical Analysis

Data were analyzed using descriptive and comparative statistical methods. Continuous variables were presented as mean, standard deviation, median, minimum-maximum, and interquartile range, while categorical variables were expressed as counts and percentages. The normality of data distribution was assessed using the Shapiro–Wilk test, histograms, Q-Q plots, as well as skewness and kurtosis values. Normally distributed variables were compared using the independent two-sample *t*-test, whereas non-normally distributed variables were analyzed with the Mann–Whitney U test. For categorical variables, the Pearson’s chi-square test or Fisher’s exact test was applied as appropriate. Diagnostic test performance was evaluated by calculating sensitivity, specificity, positive and negative predictive values, and the area under the receiver operating characteristic curve (AUC). All analyses were performed using SPSS version 23.0, with a *p*-value of <0.05 considered statistically significant. All graphical visualizations were generated using the Python programming language (version 3.10, Python Software Foundation, Wilmington, DE, USA) with the Matplotlib (version 3.8.2) and Pillow (version 10.1.0) libraries for scientific plotting and image annotation, respectively.

## 3. Results

A total of 88 patients diagnosed with endometrioid-type endometrial cancer were included in this study. The mean age of the patients was 57.6 ± 9.7 years. Most patients (72.7%) were postmenopausal. The most common presenting symptom was postmenopausal bleeding (67%), followed by abnormal uterine bleeding (26.1%). Regarding the surgical approach, 52.3% of patients underwent laparoscopy (L/S), while 38.6% underwent laparotomy (L/T), and 9.1% required conversion from laparoscopy to laparotomy. A summary of these baseline clinical and demographic characteristics is presented in Table 1.

When preoperative imaging and intraoperative assessments were compared with final pathological findings, statistically significant differences were observed (Table 2). MRI evaluation revealed that superficial myometrial invasion was detected in 58.0% of the patients. According to intraoperative frozen section analysis, this rate was 67.0%, while final pathological evaluation indicated a rate of 56.8%. Deep myometrial invasion was detected in 42.0% of cases by MRI, 33.0% by frozen section analysis, and 43.2% by final pathology. Cervical stromal involvement was identified in 3.4% of cases by frozen section, compared to 17.0% in the final pathology results. These findings suggest that both preoperative and intraoperative assessments may have limited sensitivity in detecting deep myometrial invasion and cervical stromal involvement.

**Figure 2:**
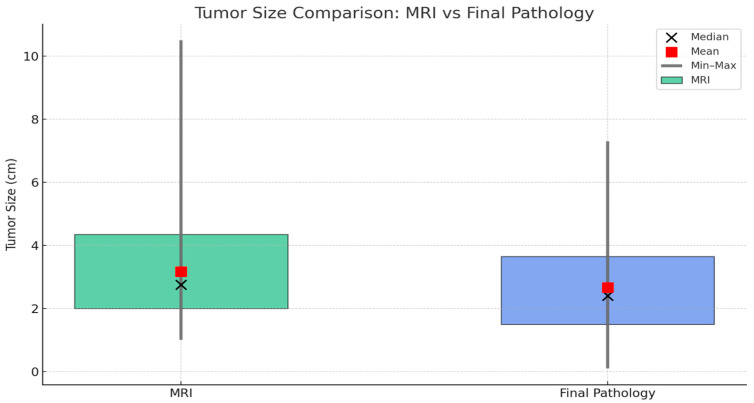
Comparison of tumor sizes measured by MRI and final pathology. Green boxes represent MRI measurements, and blue boxes represent final pathology. Boxes indicate the interquartile range (Q1–Q3), black circles show the median, red squares represent the mean, and vertical lines denote the minimum and maximum values.

Tumor sizes were evaluated using both preoperative MRI and final pathology findings (Figure 2). The mean tumor diameter measured by MRI was 3.17 ± 1.82 cm (range: 1.0–10.5 cm; median: 2.75 cm, IQR: 2.0–4.35 cm), while the mean pathological tumor diameter was 2.66 ± 1.60 cm (range: 0.1–7.3 cm; median: 2.4 cm, IQR: 1.5–3.65 cm). This difference indicates that preoperative MRI tends to slightly overestimate tumor size, which may be explained by contrast enhancement exaggerating the lesion margins and apparent tumor boundaries in dynamic sequences.

**Figure 3:**
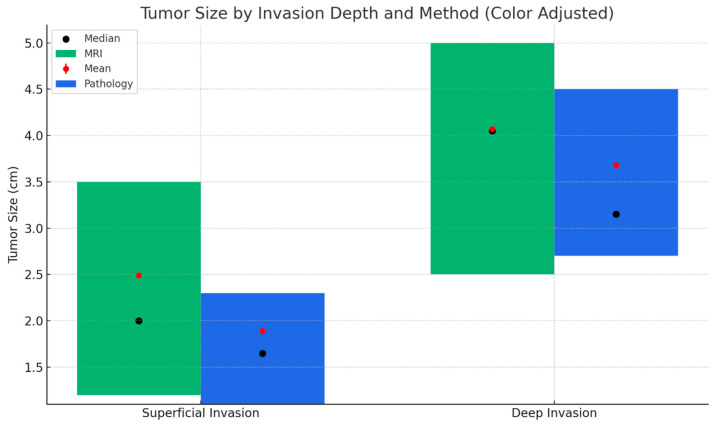
Tumor size distribution based on MRI and final pathology by depth of myometrial invasion. This figure compares tumor size measurements obtained via MRI (green) and final pathology (blue) across superficial and deep myometrial invasion groups. Bars represent the interquartile range (Q1–Q3), black dots indicate the median, and red dots represent the mean tumor size. Tumor sizes were significantly larger in the deep invasion group compared to the superficial group, according to both MRI and pathological measurements.

When tumor diameters were compared according to the depth of myometrial invasion, a statistically significant difference was observed in both MRI and final pathology measurements (Figure 3). Tumors with deep myometrial invasion were significantly larger than those with superficial invasion (*p* < 0.001), supporting the close relationship between tumor size and the extent of myometrial infiltration in endometrioid-type endometrial carcinoma.

Significant differences were observed across several variables, including MRI and frozen section assessments, cervical stromal involvement, and tumor grade, when compared according to the depth of final myometrial invasion (Table 3). Among patients with superficial invasion on final pathology, 88.0% were also reported as having superficial invasion by preoperative MRI; however, this concordance dropped to 18.4% in the deep invasion group (*p* < 0.001). The accuracy of frozen section analysis in detecting superficial invasion was 98.0%, whereas it was only 26.3% in the deep invasion group (*p* < 0.001). Cervical stromal involvement and higher tumor grade were significantly associated with deep myometrial invasion (*p* < 0.001 and *p* = 0.001, respectively), consistent with the increased aggressiveness of tumors demonstrating deeper infiltration (Table 3).

**Figure 4:**
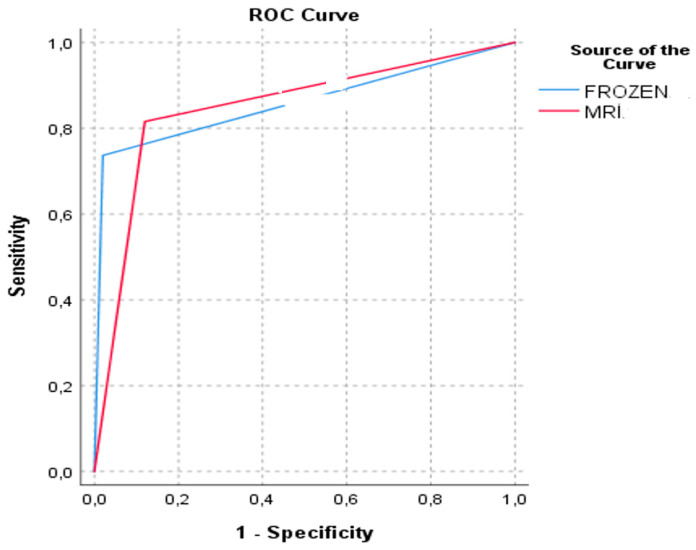
Receiver operating characteristic (ROC) curves comparing the diagnostic performance of MRI and intraoperative frozen section in detecting myometrial invasion.

When evaluating the diagnostic performance of MRI and frozen section assessments in predicting myometrial invasion, MRI demonstrated a sensitivity of 81.6%, specificity of 88.0%, positive predictive value (PPV) of 83.8%, and negative predictive value (NPV) of 86.3%. Frozen section analysis showed a sensitivity of 73.7% and a specificity of 98.0%. The area under the receiver operating characteristic curve (AUC) was 0.848 for MRI and 0.858 for frozen section evaluation. These findings are summarized in Table 4**,** and the comparative ROC curves are illustrated in Figure 4.

## 4. Discussion

In the literature, numerous studies have examined the effectiveness of MRI in determining the depth of myometrial invasion in endometrial cancer. A study evaluating the diagnostic accuracy and staging of endometrial carcinoma with MRI reported an overall diagnostic accuracy of 74%, demonstrating a significant correlation between histopathological findings and myometrial invasion as well as cervical extension. The study concluded that MRI provides high accuracy in the diagnosis and staging of endometrial carcinoma, playing a crucial role in treatment planning [20]. Furthermore, various studies have shown that MRI has a high accuracy rate in predicting myometrial invasion and is an effective method for assessing other prognostic factors such as tumor size and histological type [21,22]. In our study, the sensitivity and specificity of MRI for assessing myometrial invasion were calculated as 81.6% and 88.0%, respectively, consistent with the literature, supporting its value as a reliable diagnostic tool.

In addition to magnetic resonance imaging, intraoperative frozen section examination also emerges as an effective diagnostic tool for evaluating myometrial invasion and may assist in determining the need for surgical staging [23]. However, there are conflicting data in the literature regarding the concordance between frozen section and final pathology [24]. A meta-analysis comprising 16 studies and 2567 patients diagnosed with endometrial cancer demonstrated that intraoperative frozen section had a sensitivity of 75% and specificity of 92% in determining myometrial invasion [25]. Another study in early-stage endometrial cancer reported an 82% concordance between FS and final pathology [26]. In our study, superficial myometrial invasion identified on frozen section was confirmed in 98.0% of cases upon final pathology, while deep invasion was concordant with the final pathology in 73.7% of cases. These findings indicate that frozen section examination has a high specificity (98.0%) for assessing myometrial invasion and can be reliably used in intraoperative decision-making. However, its lower sensitivity (73.7%) compared to MRI suggests that the depth of invasion may not be accurately determined in some cases. Our results are consistent with the literature, supporting the use of frozen section evaluation to guide surgical decisions, especially regarding intraoperative lymphadenectomy.

While the frozen section technique offers high specificity, its relatively lower sensitivity indicates that it should be used in conjunction with MRI for more accurate assessment. In this context, the primary aim of our study was to evaluate the diagnostic performance of magnetic resonance imaging and intraoperative frozen section in determining the depth of myometrial invasion, and to compare these methods with the final histopathological evaluation, which served as the reference standard. In a study by Kisu et al., the accuracy, sensitivity, and specificity of MRI in detecting myometrial invasion were reported as 65.8%, 58.8%, and 88.5%, respectively, whereas frozen section demonstrated higher values for the same parameters (90.1%, 90.6%, and 88.5%, respectively), suggesting superior sensitivity of frozen section compared to MRI [27]. Similarly, large-scale investigations—including the Swedish Gynecologic Cancer Group study of 1401 patients—have reported high diagnostic accuracy for frozen section, with sensitivity and specificity values around 90% and 92%, respectively [28]. These findings, consistent across multiple reports, confirm that frozen section offers high specificity and that its use alongside MRI may enhance diagnostic accuracy in surgical decision-making [29].

In our study, both methods yielded results comparable to those reported in the literature for the assessment of myometrial invasion. MRI demonstrated superior sensitivity compared to frozen section examination (81.6% vs. 73.7%), indicating its greater effectiveness in predicting deep invasion, while the high specificity of frozen section (98.0%) made it more reliable for confirming superficial invasion. These findings are consistent with the existing literature and support the combined use of MRI and frozen section examination to more accurately assess the depth of myometrial invasion, thereby facilitating more effective surgical planning.

The depth of myometrial invasion in endometrial cancer has been shown to be closely associated with clinical and morphological parameters, particularly patient age and tumor size [30]. In our study, patients of advanced age were found to have deeper myometrial invasion and larger tumor diameters, and both variables were shown to be significantly predictive of invasion depth. These findings are consistent with the key prognostic factors identified in the literature and provide clinically valuable insights into the biological behavior of the disease [31,32,33,34,35].

Sentinel lymph node (SLN) biopsy is a widely accepted technique for lymphatic staging in endometrial cancer, but its application can be limited in some centers due to mapping failure. When mapping is unsuccessful—particularly in patients at high or high-to-intermediate risk—current guidelines recommend side-specific lymphadenectomy [15]. In such cases, pelvic MRI provides valuable preoperative information on prognostic parameters, especially myometrial invasion depth, and can assist surgical planning. The 2021 ESGO/ESTRO/ESP guidelines also emphasize that intraoperative frozen section has limited reproducibility and may compromise tissue quality for molecular testing [15]. Consistent with these observations, our study found that preoperative pelvic MRI demonstrated slightly higher diagnostic accuracy than intraoperative frozen section in assessing myometrial invasion.

One of the main strengths of this study is the direct comparison between preoperative MRI and intraoperative frozen section results with final histopathological findings. The inclusion of only patients with endometrioid-type endometrial cancer provided a homogeneous cohort for evaluating myometrial invasion. However, the retrospective design may introduce selection bias, and variations in pathological assessment criteria among institutions could affect the generalizability of results. Additionally, interobserver variability among radiologists and pathologists was not evaluated, which may have influenced the consistency of MRI and frozen section interpretations. The relatively small sample size also limits the statistical power, especially for subgroup analyses. Future prospective studies with larger cohorts are needed to confirm these findings and strengthen their clinical applicability.

## 5. Conclusions

Since the majority of endometrial cancer cases are evaluated preoperatively, preoperative imaging plays a pivotal role in facilitating accurate staging, optimizing surgical planning, and guiding patient counseling. Deep myometrial invasion is directly correlated with the risk of pelvic lymph node metastases; therefore, MRI findings may help predict the potential need for pelvic lymphadenectomy before surgery. In cases where intraoperative frozen section examination is not available or limited, MRI findings—considered alongside tumor histology and grade—may assist in surgical decision-making regarding lymphadenectomy. Moreover, MRI provides valuable preoperative information that allows for stratification of patients into low- and high-risk groups based on biopsy results, supporting a tailored surgical approach. Integrating both MRI and frozen section assessment into the clinical workflow can enhance intraoperative decision-making and improve individualized patient management.

## Figures and Tables

**Table 1:** Clinical and Demographic Characteristics of the Included Patients.

Variable	Value
Age, mean ± SD, (years)	57.57 ± 9.65
BMI, mean ± SD, (kg/m^2)^	32.33 ± 6.74
Comorbidities *n* (%) Absent Present	46 (52.3) 42 (47.7)
Menopausal Status *n* (%) Premenopausal Postmenopausal	24 (27.3) 64 (72.7)
Preoperative Complaints *n* (%) None Abnormal Uterine Bleeding Pelvic Pain Postmenopausal Bleeding Increased Endometrial Thickness Vaginal Discharge	2 (2.3) 23 (26.1) 1 (1.1) 59 (67.0) 2 (2.3) 1 (1.1)
Ultrasound Findings *n* (%) Normal Increased Endometrial Thickness Intrauterine Mass Intracavitary Fluid Irregular Endometrium	11 (12.5) 33 (37.5) 29 (33.0) 2 (2.3) 13 (14.8)
Surgical Approach *n* (%) Laparoscopy (L/S) Laparotomy (L/T) Conversion from L/S to L/T	46 (52.3) 34 (38.6) 8 (9.1)

**Table 2:** Surgical and Histopathological Evaluation Results in Patients with Endometrioid-Type Endometrial Cancer.

Assessment Parameter	Category	n	%
MRI Myometrial Invasion Depth	Superficial invasion	51	58.0
	Deep invasion	37	42.0
Frozen Section Myometrial Invasion Depth	Superficial invasion	59	67.0
	Deep invasion	29	33.0
Frozen Section Cervical Stromal Involvement	Absent	85	96.6
	Present	3	3.4
Final Pathology—Myometrial Invasion	Superficial invasion	50	56.8
	Deep invasion	38	43.2
Final Pathology—Cervical Stromal Involvement	Absent	73	83.0
	Present	15	17.0

**Table 3:** Comparison of Preoperative, Intraoperative, and Postoperative Variables According to Final Myometrial Invasion (MI) Groups.

	Final Pathology Myometrial Invasion Depth	Superficial Invasion		Deep Invasion		
		n	%	n	%	*p*-Value
**MRI Myometrial Invasion Depth**	Superficial invasion	44	88.0	7	18.4	<0.001 ^b^
	Deep invasion	6	12.0	31	81.6	
**Frozen Section Myometrial Invasion Depth**	Superficial invasion	49	98.0	10	26.3	<0.001 ^b^
	Deep invasion	1	2.0	28	73.7	
**Frozen Section Cervical Stromal Involvement**	Absent	49	98.0	36	94.7	0.576 ^a^
	Present	1	2.0	2	5.3	
**Final Pathology—Cervical Stromal Involvement**	Absent	48	96.0	25	65.8	<0.001 ^b^
	Present	2	4.0	13	34.2	
**Final Tumor Grade**	Grade 1	42	84.0 ^x^	18	47.4 ^y^	0.001 ^a^
	Grade 2	7	14.0 ^x^	16	42.1 ^y^	
	Grade 3	1	2.0 ^x^	4	10.5 ^x^	

^a^ Fisher’s Exact Test; ^b^ Pearson Chi-Square Test; ^x^, ^y^: In tables with multiple categories, when *p*-values are significant, different superscript letters indicate statistically significant differences between column percentages.

**Table 4:** Diagnostic Performance of Magnetic Resonance Imaging and Frozen Section Analysis in Assessing Myometrial Invasion Depth.

		Final Pathology Myometrial Invasion Depth								
		Superficial Invasion			Deep Invasion			AUC	AUC Difference	*p*-Value
		n	NPV (%)	Specificity (%)	n	PPV (%)	Sensitivity (%)			
**MRI Myometrial Invasion Depth**	**Superficial invasion**	44	86.3	88.0	7	13.7	18.4	0.848	0.01 (−0.103–0.082)	0.823
	**Deep invasion**	6	16.2	12.0	31	83.8	81.6			
**Frozen Section Myometrial Invasion Depth**	**Superficial invasion**	49	83.1	98.0	10	16.9	26.3	0.858		
	**Deep invasion**	1	3.4	2.0	28	96.6	73.7			

## Data Availability

The data that support the findings of this study are available from the corresponding author upon reasonable request.

## Abbreviations

AUC	Area Under the Curve
BMI	Body Mass Index
EC	Endometrial Cancer
ESGO	European Society of Gynecological Oncology
ESP	European Society of Pathology
ESTRO	European Society for Radiotherapy and Oncology
FIGO	International Federation of Gynecology and Obstetrics
FS	Frozen Section
L/S	Laparoscopy
L/T	Laparotomy
LVSI	Lymphovascular Space Invasion
MRI	Magnetic Resonance Imaging
NPV	Negative Predictive Value
PPV	Positive Predictive Value
SD	Standard Deviation
